# Study on Flexural Performance of Recycled Aggregate Concrete Beams Incorporating Glazed Hollow Beads

**DOI:** 10.3390/ma18112435

**Published:** 2025-05-23

**Authors:** Jingguang Hou, Yuanzhen Liu, Xiangzheng Li, Zhaoxu Wang

**Affiliations:** College of Civil Engineering, Taiyuan University of Technology, Taiyuan 030024, China

**Keywords:** recycled aggregate concrete, glazed hollow beads, flexural behavior, ultimate limit state, serviceability limit state, deformability, stiffness analytical method

## Abstract

Recycled aggregate concrete incorporating glazed hollow beads (GHBRC) achieves the dual objectives of energy conservation and emission reduction by combining recycled coarse aggregate with glazed hollow bead aggregate, aligning with the construction industry’s “dual-carbon” goals for the development of low-carbon concrete. This study systematically investigates the flexural performance of GHBRC beams to establish calculation formulas for ultimate limit state bearing capacity and serviceability limit state verification. Six full-scale GHBRC beams were tested under simply supported conditions with two-point symmetric mid-span loading. Three critical variables (concrete composition, longitudinal tensile reinforcement ratio, and stirrup reinforcement configuration) were examined. Experimental results indicate that GHBRC beams exhibit failure modes consistent with conventional concrete beams, confirming the validity of the plane section assumption. At identical reinforcement ratios, GHBRC beams demonstrated a 3.1% increase in ultimate bearing capacity and an 18.78% higher mid-span deflection compared to ordinary concrete beams, highlighting their superior deformation performance. Building on methodologies for conventional concrete beams, this study recalibrated key short-term stiffness parameters using a stiffness analytical method and proposed a computational model for mid-span deflection prediction. These findings provide theoretical and practical foundations for optimizing the structural design of GHBRC beams in alignment with sustainable construction objectives.

## 1. Introduction

With the promulgation of China’s “carbon peaking and carbon neutrality” strategic objectives, green low-carbon construction has emerged as a pivotal direction for energy conservation and emission reduction in the building sector. Recycled aggregate concrete (RAC) is a new type of construction material produced by partially or completely replacing natural aggregates with recycled coarse aggregate (RCA) produced by crushing construction waste concrete [1,2,3,4,5,6]. This technology system has a double environmental benefit: from the input side, it can alleviate the excessive consumption of natural sand and gravel resources, and from the output side, it can effectively reduce the land occupation and environmental pollution caused by construction waste landfill. Taking Shanghai’s engineering practice as an example, when the replacement rate of recycled aggregate reaches 10%, carbon emission reduction of more than 120,000 tons per year can be achieved, and its carbon sequestration efficiency is equivalent to maintaining the annual carbon sink level of 2820 hectares of urban green space or 6.6 million trees [7]. Recycled aggregate concrete incorporating glazed hollow beads (GHBRC), which synergistically combines recycled coarse aggregates with glazed hollow bead aggregates, serves dual purposes of thermal insulation and solid waste utilization [8]. This innovative material exemplifies low-energy consumption building materials under the dual-carbon framework, simultaneously addressing energy efficiency and emission mitigation requirements.

Glazed hollow beads, characterized by their lightweight, porous structure and low thermal conductivity, are high-performance inorganic insulation materials widely used in building insulation mortars [9]. Zhu et al. [10] investigated the thermal properties of recycled aggregate concrete (RAC) and recycled concrete blocks. The thermal conductivity of RAC was investigated using orthogonal tests considering the effects of four factors. In addition, the thermal conductivity of large particle recycled aggregate concrete and recycled brick concrete was tested at different recycled aggregate replacement rates. The shape, gradation, and volume doping of GHBs significantly affect the heat transfer properties of the composites, such as the thermal conductivity modulation mechanism, which can be obtained by the random distribution model and the Levy formula for two-phase materials [11,12]. Furthermore, the pre-soaked GHB effectively reduces the chloride ion diffusion coefficient of RAC under cracking conditions by stimulating the dual mechanism of filling pores with hydration products and adsorbing corrosive media, and the empirical relationship among resistivity, electric current, and Cl- diffusion coefficient is clarified [13,14]. At the microscopic level, GHB optimizes the pore distribution of RAC, inhibits damage propagation under high temperature load coupling, and improves thermal stability and structural durability [15,16].The incorporation of recycled aggregates and glazed hollow beads fundamentally alters the physical–mechanical characteristics of GHBRC compared to conventional concrete. Another research introduces a novel composite column—thin-walled square glazed hollow beads recycled aggregate concrete-filled double-skin steel tubular (GRCFDST) column with an inner circular hollow section [16]. Axial compression tests and numerical analyses were performed on 12 specimens to evaluate the behavior of these columns under 100% recycled coarse aggregate replacement. Ultimate strength increased with higher width-to-thickness ratios and hollow ratios but decreased with elevated GHB dosages. Ductility improved with larger hollow ratios and GHB content but declined with greater width-to-thickness ratios.

As primary horizontal load-bearing elements in building structures, beams account for approximately 30% of total concrete consumption while imposing less stringent mechanical requirements compared to vertical components like columns and shear walls. This characteristic positions GHBRC beams as an ideal application for recycled aggregate concrete. Notably, beam design in reinforced concrete frame structures predominantly focuses on flexural capacity calculations and deformation verification—critical indicators governing structural safety and serviceability. In previous years, scientists have studied the flexural properties of recycled concrete beams [17,18,19]. Bending performance studies of recycled concrete beams have shown that their mechanical properties are significantly affected by the recycled aggregate (RCA) replacement rate and the reinforcement method. For example, Sojobi et al. [20] found that carbon fiber-reinforced composite laminates could effectively increase the load-bearing capacity and ductility of recycled concrete beams through four-point bending tests and optimized the reinforcement configuration without grooving; its strength and ductility efficiency were quantitatively evaluated by equations. However, Dawood et al. [21] showed that as the percentage of natural aggregate replaced by RCA increased, the compressive and flexural strengths and ultimate loads of the beams decreased, but the crack spacing decreased and the ductility increased, which was attributed to the fragility of the transition zone at the interface between the RCA and the cementitious matrix. Avram et al. [22] presented the behavior of kaolinite matrix under bending action, illustrating the expansion of microcracks in SEM images of the failed surface. Ajdukiewicz et al. [19] reported 10–25% higher serviceability-stage deflections and 30–50% greater ultimate-stage deflections in recycled concrete beams versus conventional counterparts. Experimental investigations by Mukai et al. [23] on the flexural behavior of recycled aggregate concrete beams demonstrated that, at a reinforcement ratio of 1.4%, the mid-span deflection under ultimate limit state conditions exhibited a progressive increase with higher recycled aggregate replacement ratios. Conversely, at an elevated reinforcement ratio of 3.3%, the deflection showed minimal sensitivity to variations in recycled aggregate replacement levels.

Significant research advancements have been achieved in thermo-structural components utilizing GHBRC. Further studies on GHBRC components—specifically shear walls and columns—have been documented, yet investigations into GHBRC beams remain notably limited. The incorporation of glazed hollow beads effectively mitigates adverse effects induced by recycled aggregates [24], reducing beam self-weight while enhancing ductility and overall seismic performance. In addition, shrinkage of concrete creates tensile stresses due to confinement, which can easily lead to cracking of the concrete, thus affecting the safety and durability of the structure. Therefore, concrete shrinkage plays an important role in the design of the service limit state of structural members [25]. Taking concrete beams as an example, when NCA is completely replaced by RCA (rRCA = 100%), the bending deformation of the beams under long-term loading caused by shrinkage is about 24.3% greater than that of beams made of 100% NCA [11]. Zhang et al. [26] calculated the shear capacity of glass bead insulated concrete beams using the “truss-arch” calculation model by modifying the constitutive relationship and softening coefficient. Kong et al. [27] determined shear tests on 38 lightweight aggregate concrete beams with shear-to-span ratios in the range of 1–3 and strength class LC25 and investigated the effect of eight different types of web reinforcement on the cracking load, ultimate capacity, and deformation of the beam members. The ACI 318-71 [28] specification was used to calculate the load carrying capacity of each bar. Yang et al. [29] experimentally investigated the shear behavior of 16 LC35 lightweight aggregate concrete beams to determine the effect of concrete strength class, shear-to-span ratio, and span-to-height ratio on the shear performance of deep beams. ACI, CSA, EC2, and Tan and Cheng modeling approaches were used for the theoretical analysis and the computational results of these four modeling approaches were compared with the experimental results. Therefore, there is a need to study the properties of recycled aggregate of thermal insulation concrete beams.

In addition, current design methodologies for GHBRC structures rely on empirical amplification factors applied to conventional concrete formulas [30,31,32], lacking mechanistic considerations for glazed hollow beads and recycled aggregates. Therefore, in this paper, recycled aggregate is combined with glazed hollow bead aggregate to develop a type of glazed hollow bead recycled concrete that combines energy conservation, emission reduction, and structural performance. The bending damage mode, load-bearing capacity, and deformation characteristics of glazed hollow bead recycled concrete beams were systematically investigated through foot-scale beam tests. Based on the theory of traditional recycled concrete beams, the influence coefficients of the substitution rate of recycled concrete and the admixture of glazed hollow beads were introduced to establish the formula for calculating the bending capacity of the normal cross-section of glazed hollow bead recycled concrete beams. And by correcting the key parameter of short-term stiffness, a highly accurate calculation model of mid-span deflection was proposed, which provides a reliable theoretical basis for the application of the project. It is found that the deflection of recycled concrete beams decreases significantly with the increase in the reinforcement rate, and a design method is proposed to optimize the deflection performance by adjusting the longitudinal reinforcement rate within the range of the appropriate reinforcement, which solves the problem of excessive deflection of this type of material due to its high ductility, and provides a new way to balance the load-bearing and deflection requirements in actual projects.

## 2. Experimental Program

### 2.1. Design of Specimens

**Figure 1:**
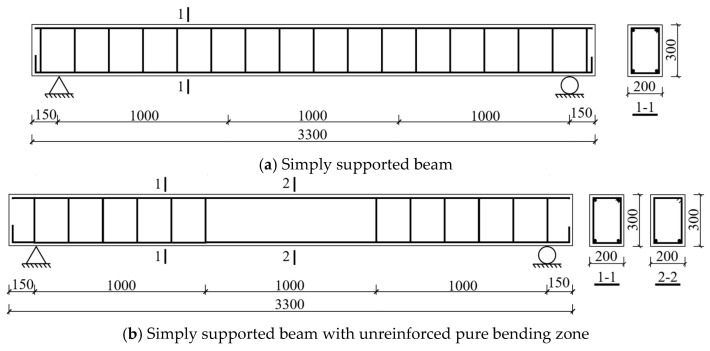
Specimen dimensions and reinforcement details.

To systematically investigate the influence mechanisms of key parameters (including recycled coarse aggregate replacement ratio, glazed hollow beads content, longitudinal tensile reinforcement ratio, and stirrup reinforcement configuration) on the mechanical behavior of concrete beams, this study designed and fabricated six full-scale beam specimens (cross-sectional dimensions: 200 mm × 300 mm; total length: 3300 mm) based on the under-reinforced beam design principle. The specimen set comprised one conventional concrete control beam, one conventional concrete incorporating glazed hollow beads and four GHBRC beams, adopting a simply supported beam system with a clear span of 3000 mm. Detailed material parameters and reinforcement configurations are provided in the mix proportion data (Table 1) and reinforcement detailing diagrams (Figure 1).

### 2.2. Experimental Loading and Measurement

#### 2.2.1. Loading Protocol

**Figure 2:**
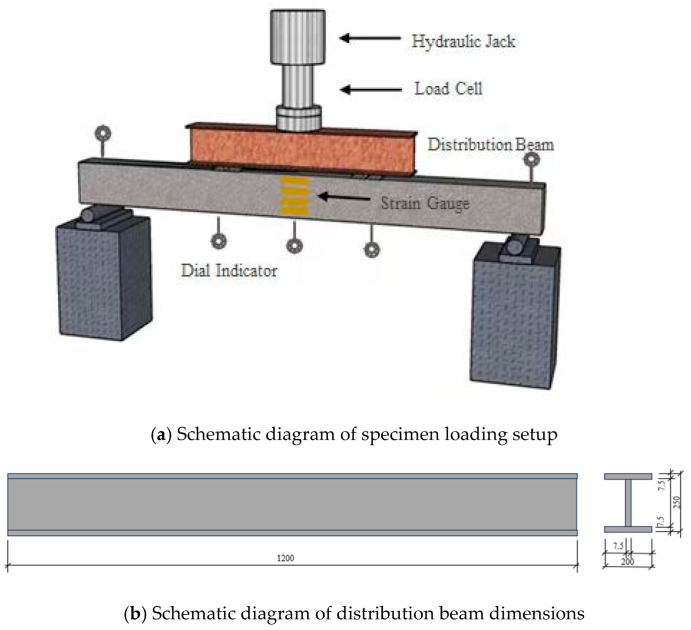
Schematic diagram of experimental equipment.

The experimental operations were strictly performed in accordance with the Chinese Standard for Test Methods of Concrete Structures GB 50152 [33], utilizing a two-point concentrated symmetrical synchronous graded loading scheme. Prior to formal loading, a pre-load of 10 kN (approximately 70% of the calculated cracking load) was applied to activate measurement instruments to normal working conditions, followed by instrument zeroing until the deformation–load relationship stabilized. The beam specimen was loaded under load-controlled protocol: before reaching the serviceability limit state load, each loading increment was set at 4 kN with a 15 min holding duration; beyond this threshold, the increment was reduced to 2 kN while maintaining the same 15 min holding duration until specimen failure. Figure 2a provides a schematic illustration of the experimental loading setup, and Figure 2b provides a schematic diagram of distribution beam dimensions.

#### 2.2.2. Measurement Instruments

**Figure 3:**
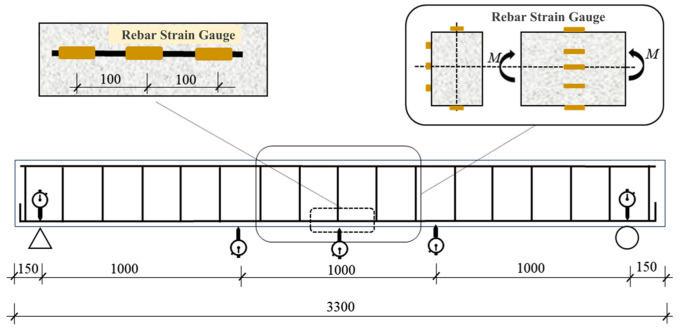
Schematic diagram of dial gauge and strain gauges.

The experimental investigation incorporated comprehensive instrumentation to analyze strain distribution and validate structural hypotheses. To assess concrete strain evolution and verify the compliance of recycled thermal insulation concrete beams with the plane section assumption, concrete strain gauges were uniformly installed at 75 mm intervals along the mid-span height (Figure 3), enabling precise monitoring of strain gradient variations across the cross-section. Simultaneously, longitudinal tensile reinforcement bars were instrumented with strain gauges through a standardized protocol involving surface preparation (derusting and protective coating removal via abrasive sanding), strain gauge degreasing, and bonding with quick-curing adhesive at pre-marked locations, as detailed in Figure 3. Dial gauges were systematically deployed at mid-span, loading points, and support regions of all beam specimens. Following bracket mounting, instruments underwent zero-adjustment and pre-loading verification to confirm operational reliability prior to formal testing, with spatial configurations illustrated in Figure 2 and Figure 3.

### 2.3. Material Properties

The cement used in this experimental study was Ordinary Portland cement (P.O 42.5). Recycled coarse aggregates were sourced from waste concrete processed through crushing and sieving procedures. Sieving tests confirmed that the recycled coarse aggregates exhibited a measured particle size distribution ranging from 4.75 mm to 20 mm, with an apparent density of 2430 kg/m^3^, crushing index of 14.48%, and water absorption of 5.28%, satisfying the specifications of Chinese Standard GB/T 25177 [34] Recycled Coarse Aggregates for Concrete. Glazed hollow bead aggregates, procured from a manufacturer in Xinyang, Henan Province of China, demonstrated a particle size of 0.5–1.5 mm, bulk density of 106 kg/m^3^, thermal conductivity of 0.043 W/(m·K), volumetric water absorption of 36%, and surface vitrification closed-pore ratio of 88%. The concrete mix proportions are summarized in Table 2, while the material properties of NC, GHBNC, and GHBRC are detailed in Table 3.

The longitudinal tensile reinforcement and construction reinforcement in this experimental study employed HRB400 steel rebars with a yield strength of 415 MPa and elastic modulus of 2.09 × 105 MPa, while the stirrups utilized HPB300 steel rebars exhibiting a yield strength of 349 MPa and elastic modulus of 2.05 × 105 MPa.

## 3. Experimental Results and Discussion

### 3.1. Failure Mode

The failure modes observed in six tested beams indicate that the GHBRC beams exhibited similar stress development and failure patterns to conventional reinforced concrete beams under loading. However, under equivalent load levels, the GHBRC beams demonstrated greater deflection magnitudes.

**Figure 4:**
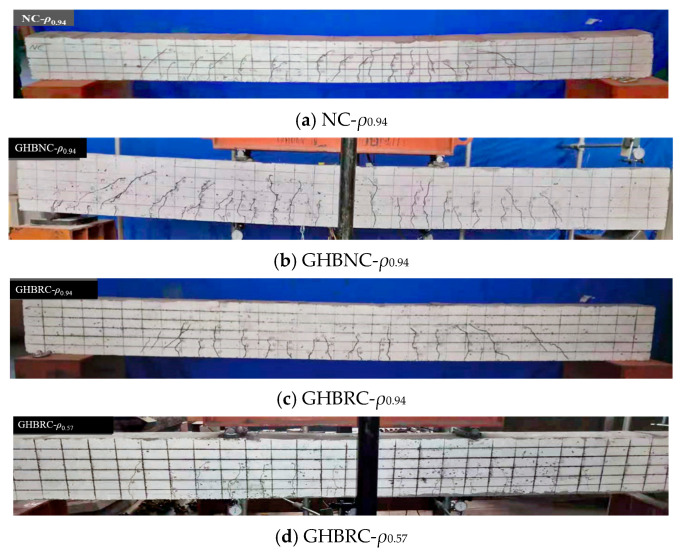

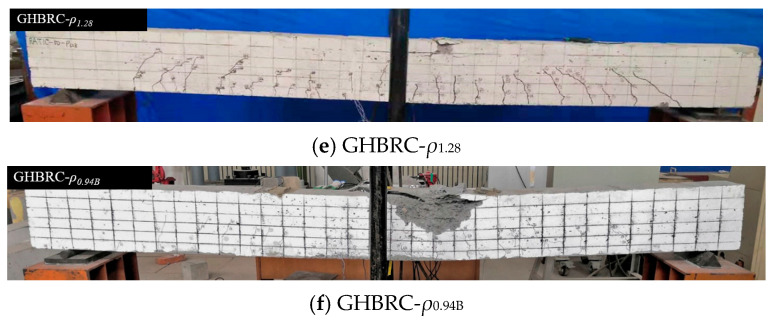
Failure modes of specimens.

For specimen GHBRC-*ρ*_0.94B_, the beam initially functioned in an elastic working state with coordinated deformation between the GHBRC and steel reinforcement. At 25.4 kN loading, initial flexural cracking occurred with a crack width of 0.08 mm. Subsequent loading induced concrete cracking in the pure bending region, characterized by vertical crack propagation along the beam height accompanied by upward neutral axis migration, followed by diagonal shear cracking. Upon reaching 48 kN, diagonal cracks emerged in the tensile zone while flexural cracks stabilized, accompanied by accelerated deflection growth. Longitudinal tensile reinforcement yielding initiated at 72.4 kN, triggering rapid deflection escalation with minimal load increment. Final failure occurred at 90.0 kN through compressive crushing of concrete in the upper region, with no further load-bearing capacity development, as illustrated in Figure 4f.

The flexural failure progression of GHBNC beams fundamentally aligns with conventional concrete beam behavior in terms of failure mechanisms and structural performance. This consistency validates the feasibility of applying established flexural theory for conventional concrete beams to analyze GHBRC beams. Figure 4 comprehensively documents the failure morphologies of all tested specimens.

### 3.2. Deflection Behavior

**Figure 5:**
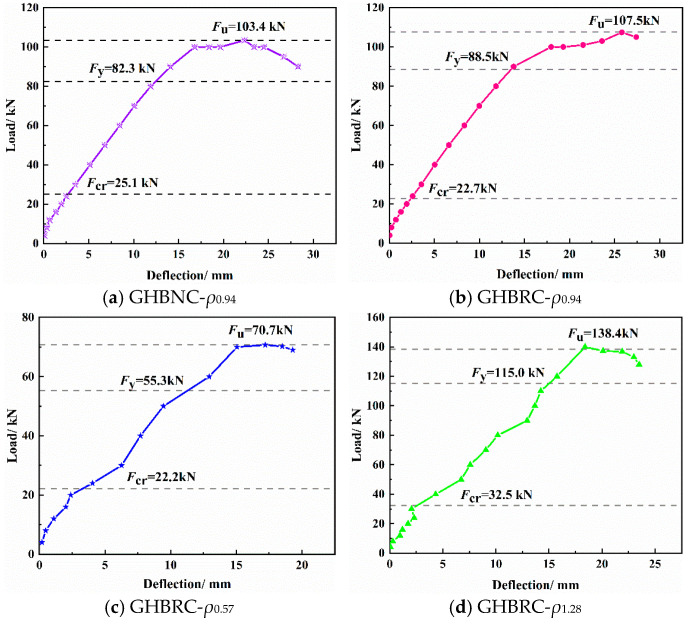

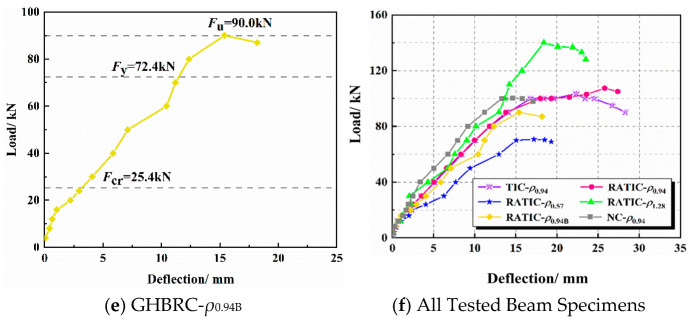
Load–deflection curve of specimens.

The load–deflection curves of the test beams were obtained through displacement correction at support locations, as shown in Figure 5. An analysis of Figure 5 indicates that the mid-span deflection development of GHBRC beams follows a pattern similar to normal concrete (NC) beams, characterized by three distinct phases, the elastic phase (pre-cracking), cracked service phase, and post-yielding phase, which is consistent with experimental observations. Specimen GHBRC-*ρ*_0.94B_, which lacked construction reinforcement in the pure bending region, exhibited a significantly shortened yield plateau and immediate failure upon steel reinforcement yielding.

During the elastic stage, cooperative load-bearing between steel and concrete resulted in high stiffness and minimal deflection. The initial stiffness followed the order of GHBRC-*ρ*_1.28_ > GHBRC-*ρ*_0.94_ > GHBRC-*ρ*_0.57_, with GHBRC-*ρ*_0.94_ slightly exceeding NC-*ρ*_0.94_, GHBNC-*ρ*_0.94_, and GHBRC-*ρ*_0.94B_. In the cracked stage, reduced curve slopes indicated degraded stiffness, yet stable deflection growth persisted until reinforcement yielding. Under identical load levels, deflection magnitudes ranked as follows for beams with matching reinforcement ratios: GHBRC beams > GHBNC beams > NC beams. Additionally, higher reinforcement ratios in GHBRC beams correlated with reduced deflections. Post-yielding, flexural stiffness decreased sharply. Specimen GHBRC-*ρ*_0.94B_ (without stirrups) failed abruptly, while others exhibited gradual deflection increases, with GHBNC-*ρ*_0.94_ achieving the maximum ultimate deflection.

Compared to NC-*ρ*_0.94_, the ultimate deflections of GHBRC-*ρ*_0.94_ and GHBNC-*ρ*_0.94_ increased by 26.6% and 35.3%, respectively. This is attributed to (1) micro-cracks inherently present within recycled aggregates, which propagated under flexural–compressive stresses, reducing compressive strength and flexural stiffness; (2) the “gas-spring effect” of uniformly distributed glazed hollow beads, enhancing structural ductility and deformation capacity. Between GHBRC beams, GHBRC-*ρ*_0.94_ and GHBRC-*ρ*_1.28_ exhibited 19% and 22% higher ultimate deflections than GHBRC-*ρ*_0.57_ due to the latter’s minimal longitudinal tensile reinforcement, which amplified deformations under equivalent loads. Post-concrete yielding, steel reinforcement dominated deformation resistance, explaining GHBRC-*ρ*_0.94_’s larger deflections than GHBRC-*ρ*_1.28_. GHBRC beams with stirrups (GHBRC-*ρ*_0.94_) showed 16.6% higher ultimate deflections than stirrup-free counterparts (GHBRC-*ρ*_0.94B_), as the absence of compressive zone stirrups in GHBRC-*ρ*_0.94B_ increased concrete stress concentrations, reducing overall post-cracking stiffness.

## 4. Flexural Bearing Capacity of Cross-Section

### 4.1. Applicability of Plane Section Assumption

The mechanical behavior of reinforced concrete members, encompassing crack initiation, propagation, deformation evolution (deflections, rotations), ultimate limit states, and failure modes, constitutes critical considerations in engineering applications. Theoretical analysis and computational methodologies for these phenomena must be grounded in cross-sectional performance evaluation. The plane section assumption serves as the foundational premise in full-range sectional analysis, necessitating validation of its applicability for GHBRC beams prior to mechanical behavior investigations.

**Figure 6:**
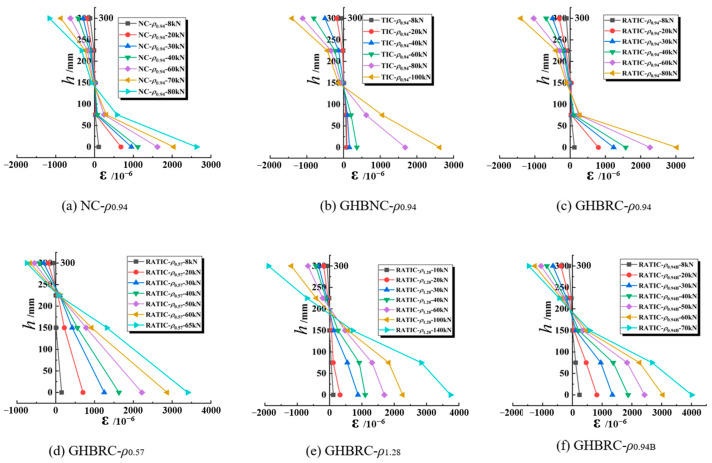
Average strain distribution of beam section height.

This study implemented concrete strain gauge arrays to measure strain distributions across mid-span sections of test beams, with the experimental results presented in Figure 6. The data demonstrate linear proportionality between concrete strain magnitudes and their distances from the neutral axis under incremental loading, confirming general compliance of GHBRC beams with the plane section assumption. This validation establishes the theoretical basis for subsequent bearing capacity calculations.

### 4.2. Analysis of Flexural Capacity of Normal Sections

#### 4.2.1. Basic Assumptions

**Figure 7:**
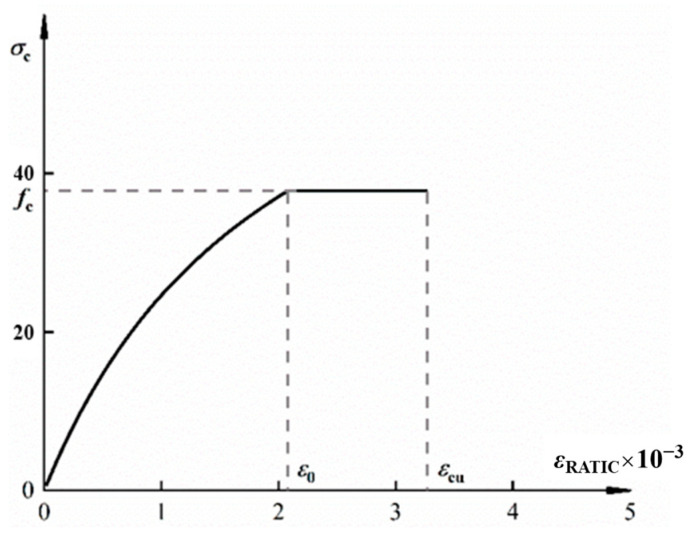
Compressive stress–strain constitutive curve of GHBRC.

In the theoretical analysis of the flexural behavior of GHBRC beams, the establishment of a quantifiable mechanical model and the assurance of engineering applicability for computational results necessitate the construction of a theoretical framework based on the following fundamental assumptions:

(1) Plane section assumption: The strain distribution across the cross-section of GHBRC beams exhibits compliance with the plane section hypothesis during flexural deformation.

(2) Neglect of tensile contribution in GHBRC: Prior experimental investigations have demonstrated that the splitting tensile strength of GHBRC is significantly reduced (approximately 38–42% compared to conventional concrete), thereby justifying the exclusion of its tensile resistance in load-bearing capacity calculations.

(3) Adoption of constitutive relationships for GHBRC: Compressive and tensile stress–strain constitutive relationships derived from the research group’s prior studies are employed. Specifically, the axial compressive stress–strain constitutive model [35] for GHBRC is expressed as follows:

(1) $$y=\left\{\begin{array}{l} \frac{3.05x}{2.05+x^{2}}(x\leq 1)\\ \frac{x}{0.849{\left(x-1\right)}^{2}+x}(x>1) \end{array}\right.$$

where $x=\varepsilon_{c}/\varepsilon_{\mathrm{cu}}$, $y=\sigma_{c}/f_{c}$, $\sigma_{c}$, and $\varepsilon_{c}$ represent the compressive stress and compressive strain of the GHBRC, respectively, while $f_{c}$ and $\varepsilon_{\mathrm{cu}}$ denote its compressive strength and compressive strain corresponding to the peak stress, respectively. The compressive stress–strain curve of GHBRC is illustrated in Figure 7, where *f*_c_ = 34.2 MPa, $\varepsilon_{0}=2.076\times 10^{-3}$, $\varepsilon_{\mathrm{cu}}=3.261\times 10^{-3}$, thereby defining the failure criterion under uniaxial compression for this material.

**Figure 8:**
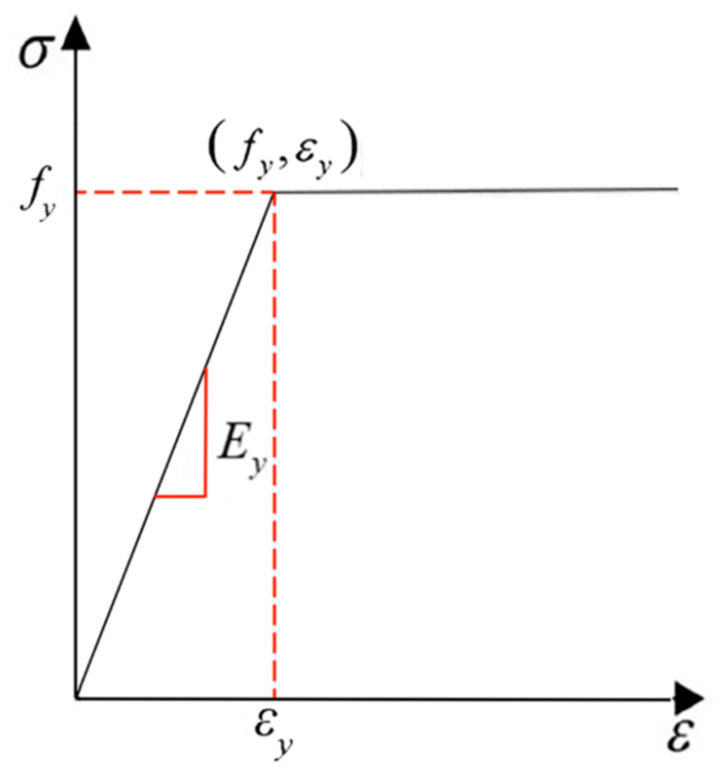
Elastic–plastic constitutive model of steel.

(4) The stress–strain constitutive model of steel reinforcement adopts an elastic–plastic relationship, as illustrated in Figure 8, with the ultimate tensile strain of the longitudinal tensile reinforcement specified as 0.01.

#### 4.2.2. Analytical Formula for Flexural Capacity of Normal Sections

**Figure 9:**
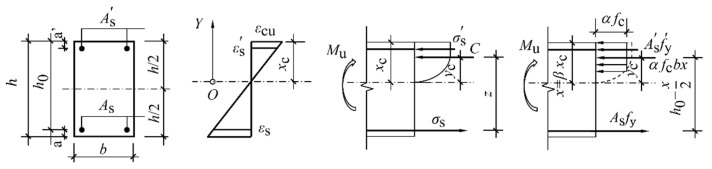
Equivalent rectangular strain diagram of beam.

Figure 9 illustrates the stress distribution diagram across the flexural section of a concrete beam. By integrating the mechanical behavior of conventional concrete beams and applying static equilibrium conditions, the following relationships (Equations (3)–(5)) are derived:

(2) $$\alpha_{\mathrm{GHBRC}}f_{\mathrm{cGHBRC}}bx+f_{y}^{\prime }A_{s}^{\prime }=f_{y}A_{s}$$

(3) $$x=\beta x_{c}$$

(4) $$M_{u}=\alpha_{\mathrm{GHBRC}}f_{\mathrm{cGHBRC}}bx(h_{0}-\frac{x}{2})+f_{y}^{\prime }A_{s}^{\prime }\left(h_{0}-a_{s}^{\prime }\right)$$

In the equations, *M*_u_ denotes the design value of the flexural capacity for the normal section, and x represents the equivalent height of the rectangular stress block in the compression zone. *α*_GHBRC_ is the equivalent rectangular stress block coefficient for GHBRC. *f*_cGHBRC_ denotes the compressive strength. By substituting Equation (3) into Equation (5), the following expression is derived:

(5) $$M_{u}=f_{y}A_{s}h_{0}-f_{y}^{\prime }A_{s}^{\prime }a_{s}^{\prime }-\frac{{\left(f_{y}A_{s}-f_{y}^{\prime }A_{s}^{\prime }\right)}^{2}}{2\alpha_{\mathrm{GHBRC}}f_{\mathrm{cGHBRC}}b}$$

In the formula, *f*_y_ represents the tensile strength of the reinforcement, while *A*_s_ denotes the area of the tensile reinforcement. The effective depth of the beam section is given by *h*_0_. The compressive strength and area of the reinforcement are represented by *f*_y_′ and *A*_s_’, respectively, indicating the distance from the resultant force point of the compressive reinforcement to the compression edge. Additionally, *b* denotes the width of the beam section.

Based on Figure 7 and Figure 9, the distances from the resultant stress in the compression zone and the total resultant force *C*_CU_ to the neutral axis can be determined as follows:

(6) $$Z=\int_{0}^{\varepsilon_{\mathrm{cu}}}\sigma_{c}\left(\varepsilon_{c}\right)\cdot b\cdot \frac{x_{c}}{\varepsilon_{\mathrm{cu}}}d\varepsilon_{c}=x_{c}\cdot b\cdot \frac{C_{\mathrm{cu}}}{\varepsilon_{\mathrm{cu}}}=k_{1}f_{\mathrm{cGHBRC}}bx_{c}$$

(7) $$y_{c}=\frac{\int_{0}^{\varepsilon_{\mathrm{cu}}}\sigma_{c}\left(\varepsilon_{c}\right)\cdot b\cdot {\left(\frac{x_{c}}{\varepsilon_{\mathrm{cu}}}\right)}^{2}\cdot \varepsilon_{c}\cdot d\varepsilon_{c}}{x_{c}\cdot b\cdot \frac{C_{\mathrm{cu}}}{\varepsilon_{\mathrm{cu}}}}=x_{c}\cdot \frac{y_{\mathrm{cu}}}{\varepsilon_{\mathrm{cu}}}=k_{2}x_{c}$$

where *k*_1_ and *k*_2_ are solely dependent on the shape of the compressive stress–strain curve of concrete. Let the stress value and height of the equivalent rectangular stress block be denoted as $\alpha_{\mathrm{GHBRC}}$, $f_{cGHBRC}$, and *x*, respectively. Based on the equivalence conditions (force equilibrium and strain compatibility), the following equation is derived from Equations (6) and (7):

(8) $$\left.\begin{array}{l} Z=\alpha_{\mathrm{GHBRC}}f_{\mathrm{cGHBRC}}bx=k_{1}f_{\mathrm{cGHBRC}}bx\\ x=2\left(x_{c}-y_{c}\right)=2\left(1-k_{2}\right)x_{c} \end{array}\right\}$$

Let $\beta_{\mathrm{GHBRC}}=x/x_{c}=2\left(1-k_{2}\right)$, then $\alpha_{\mathrm{GHBRC}}=\frac{k_{1}}{\beta_{\mathrm{GHBRC}}}=\frac{k_{1}}{2\left(1-k_{2}\right)}$. The coefficients $\alpha_{\mathrm{GHBRC}}$ and $\beta_{\mathrm{GHBRC}}$ represent the equivalent rectangular stress block parameters for GHBRC. These parameters are solely determined by the constitutive curve of GHBRC.

By treating $\alpha_{\mathrm{GHBRC}}$ and $\beta_{\mathrm{GHBRC}}$ as coefficients related to recycled aggregates and glazed hollow beads, this study investigates the differences between GHBRC beams and conventional concrete beams. Combining the experimental results, a regression analysis was performed to establish the relationship between these parameters, yielding the following findings, as shown in Table 4.

By substituting the results from Table 4 into Equation (5), the calculated flexural capacity values for the test beams in this study were obtained, as summarized in Table 5.

As shown in Table 5, the ratio of experimental values to calculated values ranges between 0.97 and 1.03, with a relative error within ±5%. Therefore, Equations (2)–(8) are applicable for calculating the flexural capacity of GHBRC beams in this study. Based on the experimental observations and data obtained from the flexural tests of GHBRC beams, it can be concluded that the flexural behavior of the test beams is similar to that of conventional concrete beams. Consequently, the flexural capacity calculation formula for the test beams was derived based on the established formulas for conventional concrete beams.

An analysis of Table 5 reveals that the incorporation of glazed hollow bead aggregates slightly enhances the flexural capacity of the test beams. When the replacement ratio of recycled coarse aggregates is 50%, the addition of 130 kg/m^3^ of glazed hollow bead aggregates increases the beam’s load-bearing capacity by 3.1%. This improvement is attributed to the sustained water absorption capability of glazed hollow beads. Since glazed hollow beads are incorporated using the water compensation method [36], they possess the ability to absorb excess free water from the freshly mixed cement paste and the interfacial transition zone surrounding the recycled coarse aggregates during mixing and hardening. This process not only enhances the stiffness of the interfacial transition zone around the recycled coarse aggregates but also improves the stiffness of the freshly mixed cement paste. Similarly to conventional concrete, increasing the longitudinal reinforcement ratio within the under-reinforced range can further enhance the beam’s load-bearing capacity.

## 5. Mid-Span Deflection Calculation Model

### 5.1. Mid-Span Deflection Analysis

As indicated in the foregoing analysis, under loading, the development of the “load-deflection” curve and the variation in sectional stiffness in GHBRC beams are similar to those of conventional reinforced concrete beams. Therefore, the stiffness calculation formula for conventional reinforced concrete beams can be referenced [33]. Among the commonly used stiffness calculation methods, the analytical stiffness method requires fewer parameters, and the material-related parameters needed in the formula can be derived from prior research findings of this research group. Consequently, the analytical stiffness method is selected to revise the short-term stiffness calculation for GHBRC, with the formula expressed as follows:

(9) $$B_{s}=\frac{E_{s}A_{s}h_{0}^{2}}{\frac{\Psi }{\eta }+\frac{\alpha_{E}\rho }{\zeta }}$$

where *E*_s_ represents the elastic modulus of the tensile reinforcement, while *A*_s_ denotes its area. The effective depth of the beam section is given by *h*_0_, and *Ψ* is the coefficient of non-uniform strain in the reinforcement. The internal force arm coefficient is represented by *η*, and *α*_E_ is the ratio of the elastic modulus of tensile reinforcement to that of concrete, with the elastic modulus of foamed glass bead concrete and foamed glass bead recycled concrete obtained from experimental measurements. Additionally, *ρ* denotes the reinforcement ratio of tensile reinforcement, and ζ is the elastic–plastic section resistance moment coefficient. The coefficients *η*, *ζ*, and *Ψ* must be determined based on experimental data.

#### 5.1.1. Internal Lever Arm Coefficient

By neglecting the tensile contribution of concrete and applying equilibrium conditions at the cracked beam cross-section, the expression for the internal lever arm coefficient can be derived as follows:

(10) $$\eta_{\mathrm{RATIC}}=\frac{M}{\varepsilon_{s}E_{s}A_{s}h_{0}}$$

Therefore, by measuring the reinforcement strain at the crack location, the internal force arm coefficient can be calculated. Here, $\varepsilon_{s}$ is taken as the maximum strain value of the reinforcement at the mid-span of the beam.

**Figure 10:**
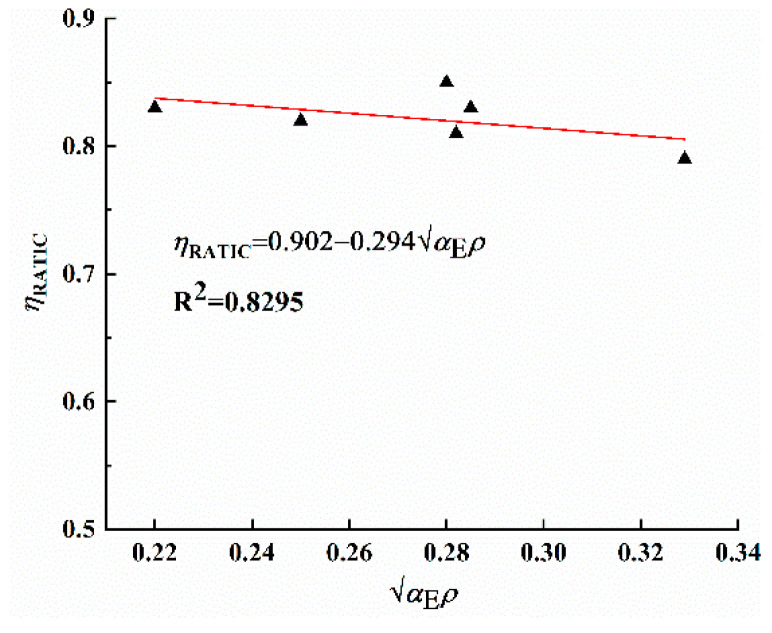
Relationship between $\eta_{\mathrm{RATIC}}$ and $\sqrt{\alpha_{E}\rho }$.

Based on the plane section assumption, the equilibrium conditions at the cracked sections were analyzed. Through regression analysis of experimentally measured parameters $\eta_{\mathrm{RATIC}}$ and $\sqrt{\alpha_{E}\rho }$ (as shown in Figure 10), the relationship between them can be established as follows:

(11) $$\eta_{\mathrm{RATIC}}=0.902-0.294\sqrt{\alpha_{E}\rho }$$

The internal lever arm coefficient of GHBRC beams is smaller than that of conventional concrete beams. Under identical conditions, the elastic modulus of GHBRC is lower than that of conventional concrete. When subjected to equivalent loads, the strain in tensile reinforcement of GHBRC beams exceeds that of conventional concrete beams. However, the internal lever arm coefficient ultimately stabilizes at a fixed value, for which $\eta_{\mathrm{RATIC}}=0.82$ is recommended.

#### 5.1.2. The Strain Non-Uniformity Coefficient of Reinforcing Steel $\psi_{\mathrm{RATIC}}$

With reference to the calculation model for the non-uniformity coefficient ψ of conventional concrete beams [37], the analytical model for determining the non-uniformity coefficient of GHBRC beams can be derived as follows:

(12) $$\psi_{\mathrm{RATIC}}=\alpha_{2}-\beta_{2}\frac{f_{\mathrm{tk}}}{\sigma_{s}\rho_{\mathrm{te}}}$$

where *f*_tk_ represents the characteristic tensile strength of foamed GHBRC concrete, while *σ*_s_ denotes the reinforcement stress at the crack location. Additionally, *ρ*_te_ refers to the effective reinforcement ratio of the beam section.

**Figure 11:**
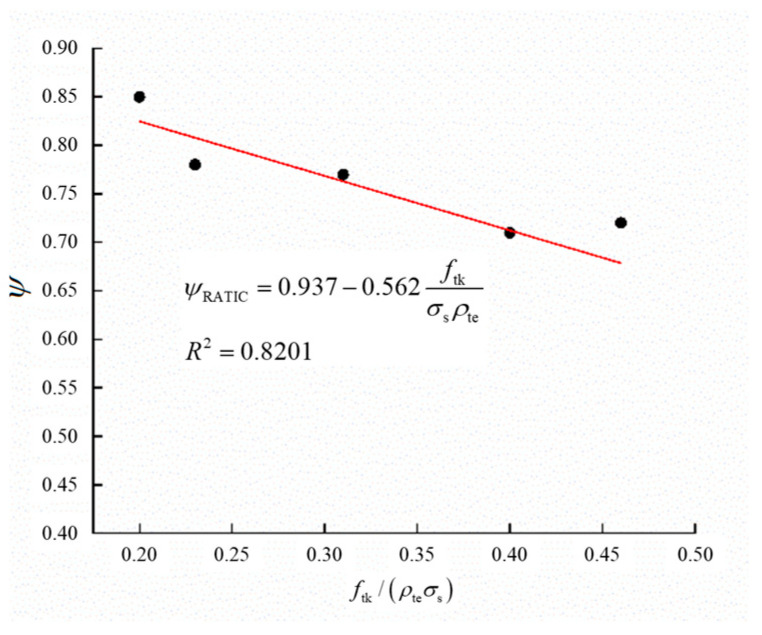
Relationship between $\psi_{\mathrm{RATIC}}$ and $f_{\mathrm{tk}}/\left(\rho_{\mathrm{te}}\sigma_{s}\right)$.

As indicated by Equation (12), the strain non-uniformity coefficient of reinforcing steel (*ψ*) exhibits strong correlation with steel stress *σ*_s_. During the loading process, however, *σ*_s_ increases proportionally with applied loads, consequently causing continuous variation in *ψ*. To determine this coefficient in practical engineering scenarios, the computation adopts *σ*_s,_ yielding the longitudinal reinforcement in the tensile zone of concrete beams. A regression analysis of *ψ* based on experimental measurements is presented in Figure 11.

Through regression analysis of experimental data, the computational model for the non-uniformity coefficient of GHBRC beams under loading conditions is formulated as follows:

(13) $$\psi_{\mathrm{RATIC}}=0.937-0.562\frac{f_{\mathrm{tk}}}{\sigma_{s}\rho_{\mathrm{te}}}$$

As revealed by Equation (13), the non-uniformity coefficient of GHBRC beams exceeds that of conventional concrete beams. Combined with the definition of the steel strain non-uniformity coefficient, this phenomenon indicates that under identical steel strain at cracked sections, GHBRC beams exhibit greater average strain in longitudinal reinforcement. This mechanical behavior demonstrates the material’s reduced tensile resistance capacity in cooperating with steel reinforcement, along with inferior bond performance between GHBRC and reinforcing bars, as evidenced in prior studies.

#### 5.1.3. The Cross-Sectional Elastoplastic Moment Resistance Coefficient $\zeta_{\mathrm{RATIC}}$

**Figure 12:**
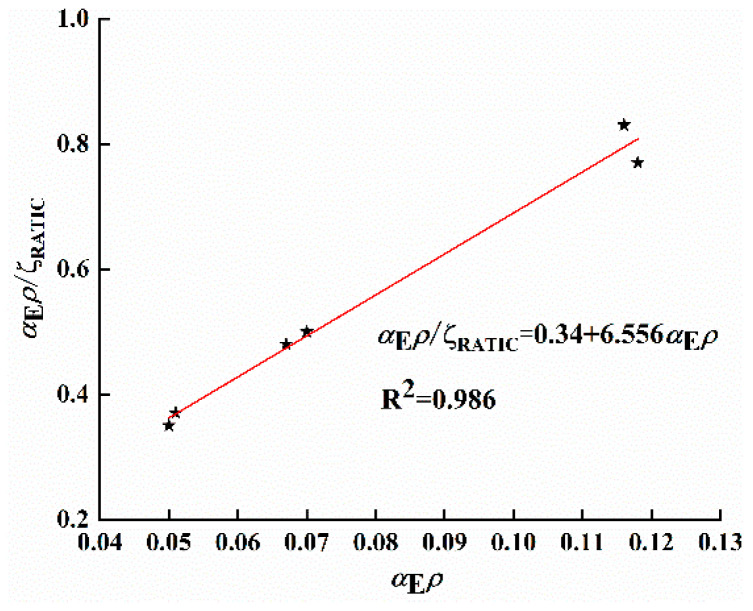
Relationship between $\alpha_{E}\rho /\zeta_{\mathrm{RATIC}}$ and $\alpha_{E}\rho$.

For design calculations of GHBRC beams, reference is made to the computational formula for conventional concrete proposed by Professor Ding [38]. Building upon regression analysis of experimental value $\zeta_{\mathrm{GHBRC}}$ and measured value $\alpha_{E}\rho$ (as shown in Figure 12), the expression for the elastoplastic moment resistance coefficient $\zeta_{\mathrm{GHBRC}}$ is formulated as follows:

(14) $$\frac{\alpha_{E}\rho }{\zeta_{\mathrm{GHBRC}}}=0.34+6.665\alpha_{E}\rho$$

A comparative analysis between the coefficient $\zeta_{\mathrm{GHBRC}}$ in Equation (14) and corresponding values specified in concrete structure design codes reveals that the elastoplastic moment resistance coefficient of GHBRC beams is lower than that of conventional concrete beams. This phenomenon is attributed to the reduced elastic modulus of GHBRC compared to conventional concrete under identical steel reinforcement modulus and reinforcement ratio. Consequently, under equivalent loading conditions, the compression zone depth at cracked sections of GHBRC beams measures smaller than conventional beams, demonstrating consistent correlation with preceding analytical results.

### 5.2. Mid-Span Deflection Calculation

Through an analysis of the experimental results from GHBRC beams, three critical parameters for calculating short-term stiffness were established: the internal lever arm coefficient ($\eta_{\mathrm{GHBRC}}$), steel strain non-uniformity coefficient ($\psi_{\mathrm{GHBRC}}$), and elastoplastic moment resistance coefficient ($\zeta_{\mathrm{GHBRC}}$), as, respectively, defined in Equations (11), (13), and (14). Incorporating these coefficients into Equation (10) yields the computational formula for short-term stiffness of GHBRC beams:

(15) $$B_{GHBRC}^{s}=\frac{E_{s}A_{s}h_{0}^{2}}{1.04\Psi_{GHBRC}+0.34+6.7\alpha_{E}\rho }$$

The mid-span deflection values of test beams are computed through structural mechanics methodology, with the analytical expression formulated as follows:

(16) $$f_{\mathrm{GHBRC}}=\frac{0.1065Ml_{0}^{2}}{B_{\mathrm{GHBRC}}^{s}}$$

Substituting Equation (15) into Equation (16) yields comparative results of the experimental versus calculated mid-span deflection values for all test beams, as tabulated in Table 6.

As evidenced in Table 6, the calculated mid-span deflection values of GHBRC beams demonstrate close agreement with experimental measurements, exhibiting a mean ratio of 0.9572, standard deviation of 0.0368, and coefficient of variation 0.038. These statistical parameters substantiate the validity and reliability of the proposed short-term stiffness formulation in fulfilling computational accuracy requirements while ensuring structural safety.

## 6. Conclusions and Remarks

The present study systematically investigates the structural performance of GHBRC beams through comprehensive experimental investigations and theoretical derivations. By examining the fundamental mechanical properties, failure mechanisms, and deformation characteristics under flexural loading, this research establishes critical relationships between material composition and structural behavior. The following conclusions can be drawn:

(1) The failure modes of GHBRC beams exhibit close resemblance to those of conventional concrete beams. The concrete strain distribution at mid-span demonstrates uniform variation along beam depth, adhering to the plane section assumption. This confirms the applicability of existing theoretical frameworks for conventional concrete beam analysis to GHBRC systems. Furthermore, the load-response progression observed in flexural tests of GHBRC beams is characterized by three distinct phases identical to conventional concrete behavior: elastic phase (pre-cracking), cracking propagation phase, and reinforcement yielding phase.
(2) The incorporation of glazed hollow bead aggregates induces a “gas-spring” mechanism within the concrete matrix, resulting in 35% greater ultimate mid-span deflection (and enhanced ductility in recycled concrete beams. Experimental data demonstrate an inverse correlation between reinforcement ratio and deformation characteristics: specimens GHBRC-*ρ*_1.28_ and GHBRC-*ρ*_0.94_ exhibit 19% and 22% reduced ultimate deflections, respectively, compared to GHBRC-*ρ*_0.57_. Consequently, strategic enhancement of the reinforcement ratio within the under-reinforced beam design range proves effective in controlling the deflection of thermal-insulating recycled concrete beams.
(3) Four fundamental postulates are established for GHBRC beams, through which the constitutive relationship between the rectangular stress block equivalence factor and aggregate composition ratios is derived. This formulation validates the rationality of flexural capacity calculation models, with experimental results demonstrating 3.1% higher ultimate flexural capacity compared to conventional concrete beams at equivalent reinforcement ratios. The quantified mechanical enhancement, coupled with its inherent thermal insulation properties, confirms the structural GHBRC in load-bearing applications while satisfying energy efficiency requirements.
(4) The three critical parameters governing short-term stiffness calculation were established through multivariate regression analysis, culminating in the formulation for GHBRC beams. Implementation of this equation enables the precise determination of mid-span deflection under incremental loading stages. Comparative analysis reveals strong agreement between the computational results and the experimental measurements. This validation confirms the robust applicability of the derived stiffness formulation for GHBRC beam systems.

In the future, based on the results of this paper, the effect of microcracks in recycled aggregates on structural defects can be further investigated by microscopy and other means to reveal the mechanism of crack propagation and realize the early prevention of structural failure. Investigate the optimum ratio of glass beads to recycled coarse aggregate and combine the synergistic effect of fibers or nanomaterials to further improve the crack resistance, impact resistance, and durability of concrete. Investigating the overall performance of glass bead-recycled concrete in framework structures, including the performance of beam–column joints and beam–shear wall synergistic forces, as well as its improvement effect on the thermal performance of buildings will help to further determine the most suitable material. The above and other aspects of research promote the development of GHBRC structures through.

## Figures and Tables

**Table 1:** Specimen number and reinforcement.

Specimen Label	*b* × *h* × *l* (mm)	Reinforcement Details			
		Tensile Reinforcement		Stirrups	Spacer Bars
		Reinforcement Ratio	Reinforcement Arrangement		
$\mathrm{NC}-\rho_{0.94}$	200 × 300 × 3300	0.94%	2C18	Φ6@200	2C12
$\mathrm{GHBNC}-\rho_{0.94}$	200 × 300 × 3300	0.94%	2C18	Φ6@200	2C12
$\mathrm{GHBRC}-\rho_{0.57}$	200 × 300 × 3300	0.57%	2C14	Φ6@200	2C12
$\mathrm{GHBRC}-\rho_{0.94}$	200 × 300 × 3300	0.94%	2C18	Φ6@200	2C12
$\mathrm{GHBRC}-\rho_{0.94B}$	200 × 300 × 3300	0.94%	2C18	Φ6@200	-
$\mathrm{GHBRC}-\rho_{1.28}$	200 × 300 × 3300	1.28%	2C22	Φ6@200	2C12

Note: NC denotes normal concrete (reference specimen), GHBNC represents conventional concrete incorporating glazed hollow beads, and GHBRC represents recycled aggregate concrete incorporating glazed hollow beads, where *ρ* indicates longitudinal tensile reinforcement ratio. Additionally, B refers to pure bending beam configuration, characterized by absence of spacer bars and stirrups in mid-span pure bending zone. NC-*ρ*_0.94_ NC-*ρ*_0.94._

**Table 2:** Mix proportions of concrete.

Classification	Relevant Ratios				Partial Composition (kg/m^3^)			
	Water/ Binder	NCA/ Water	RCA/ Cement	Sand/ Cement	Cement	Glazed Hollow Beads	Silica Fume	Water-Reducing Admixture
NC	0.52	2.50	0	1.07	484	0	36	5.70
GHBNC	0.52	2.50	0	1.07	484	169	36	5.70
GHBRC	0.52	1.25	1.20	1.07	484	169	36	5.70

Note: NCA denotes Natural Coarse Aggregate, while RCA represents recycled coarse aggregate.

**Table 3:** Physical properties of concrete.

Classification	*Ρ* (kg/m^3^)	*f*_c_ (MPa)	*E*_c_ (10^4^ N/mm^4^)	*λ* (W/(m⸱K))
NC	2500 ± 26.4	37.5 ± 0.36	3.01 ± 0.03	1.74 ± 0.02
GHBNC	1800 ± 36.1	36.2 ± 0.20	2.63 ± 0.04	0.40 ± 0.01
GHBRC	1730 ± 34.6	34.2 ± 0.26	2.36 ± 0.02	0.56 ± 0.02

Note: *Ρ* represents the density of concrete (unit: kg/m^3^), *f*_c_ denotes the compressive strength of concrete (unit: MPa), *E*_c_ corresponds to the elastic modulus of concrete (unit: MPa), and *λ* signifies the thermal conductivity of concrete (unit: W/(m⸱K)).

**Table 4:** Equivalent rectangular strain coefficient in compression zone of GHBRC.

	NC	GHBNC	GHBRC
$\alpha_{\mathrm{GHBRC}}$	1.0	0.996	0.989
$\beta_{\mathrm{GHBRC}}$	0.798	0.793	0.785

**Table 5:** Results of comparison between experimental and calculated flexural capacity of specimens.

Specimens	Flexural Capacity (kN)		*M*_u,test_/*M*_U_
	*M*_u,test_	*M*_U_	
NC-*ρ*_0.94_	100.3 ± 0.95	98.3	1.02 ± 0.01
GHBNC-*ρ*_0.94_	103.4 ± 1.79	104.4	0.99 ± 0.02
GHBRC-*ρ*_0.94_	107.5 ± 2.76	104.2	1.03 ± 0.03
GHBRC-*ρ*_0.57_	70.7 ± 0.70	72.8	0.97 ± 0.01
GHBRC-*ρ*_1.28_	138.4 ± 5.14	142.6	0.97 ± 0.04
GHBRC-*ρ*_0.94B_	93.4 ± 2.49	94.3	0.99 ± 0.03

**Table 6:** Results of comparison between experimental and calculated deflection of GHBRC.

Speciments	Load (kN)	*F*_GHBRC_ (mm)		Test/Equation (16)
		Test	Equation (16)	
	30	2.48 ± 0.05	2.70	0.92 ± 0.02
NC-*ρ*_0.94_	60	6.75 ± 0.19	7.03	0.96 ± 0.03
	90	11.20 ± 0.12	11.79	0.95 ± 0.01
	30	3.51 ± 0.13	3.58	0.98 ± 0.04
GHBNC-*ρ*_0.94_	60	8.45 ± 0.24	9.29	0.91 ± 0.03
	90	14.10 ± 0.26	15.00	0.94 ± 0.02
	30	3.56 ± 0.04	3.83	0.93 ± 0.01
GHBRC-*ρ*_0.94_	60	8.33 ± 0.34	8.50	0.98 ± 0.04
	90	13.79 ± 0.15	14.36	0.96 ± 0.01
	30	6.26 ± 0.19	6.96	0.90 ± 0.03
GHBRC-*ρ*_0.57_	60	12.04 ± 0.47	13.07	0.92 ± 0.04
	70	15.05 ± 0.16	15.68	0.96 ± 0.01
	30	2.05 ± 0.10	2.09	0.98 ± 0.05
GHBRC-*ρ*_1.28_	60	7.58 ± 0.19	7.43	1.02 ± 0.03
	90	12.94 ± 0.13	12.81	1.01 ± 0.01
	30	4.08 ± 0.14	4.00	1.02 ± 0.04
GHBRC-*ρ*_0.94B_	60	10.43 ± 0.31	11.46	0.91 ± 0.03
	90	15.40 ± 0.57	15.71	0.98 ± 0.04

## Data Availability

The original contributions presented in this study are included in the article. Further inquiries can be directed to the corresponding author.
